# Eliciting health beliefs: Difficulties and solutions

**DOI:** 10.1371/journal.pone.0347922

**Published:** 2026-05-28

**Authors:** Xiaoxue Sherry Gao, Rong Rong

**Affiliations:** Department of Resource Economics, University of Massachusetts Amherst, Amherst, Massachusetts, United States of America; Virginia Commonwealth University, UNITED STATES OF AMERICA

## Abstract

Despite recent progress in the development of incentive-compatible belief elicitation methods, some unique challenges arise when researchers apply some methods to elicit one’s beliefs about their own health status. First, the unavailability or unverifiability of one’s own health outcomes to incentivize the elicitation procedure. Second, the opportunity to hedge against adverse health outcomes by increasing the associated monetary payoffs in an incentivized elicitation task. To solve these issues, we elicit participants’ beliefs about their close peers as proxies for their beliefs about themselves. We first collected the health outcomes of others with similar health status using a pre-experiment survey. Then, we used the surveyed outcomes to incentivize the belief elicitation procedure in the laboratory experiment stage. We conclude that the elicited beliefs are reflective of their beliefs about their own health status because the elicited beliefs using our proposed method are highly correlated with the participants’ own past health outcomes.

## 1. Introduction

Health beliefs are central for understanding health behaviors. For example, beliefs about susceptibility to communicable diseases can influence individuals’ willingness to be vaccinated. Understanding how such beliefs shape behavior is therefore essential for designing information-based policy interventions to promote public health. Like other subjective beliefs, health beliefs are unobservable in natural settings and must be elicited to study their behavioral consequences.

Substantial progress has been made in developing and testing incentive-compatible procedures for eliciting subjective beliefs [1–3]. However, when applied to elicit beliefs about one’s own health status, there are some unique challenges. This study identifies such challenges and proposes practical solutions. We illustrate our approach using the Quadratic Scoring Rule (QSR; [4–10]) to elicit influenza vaccination–related beliefs. Although we focus on the QSR as an illustrative example, the challenges we identify and the solutions we propose apply broadly to many other popular incentive-compatible elicitation methods.

To provide context and motivation, we elicit vaccination-related beliefs to examine how they affect influenza vaccination decisions among undergraduate students at the University of Massachusetts Amherst (UMass Amherst). Our framework is the Health Belief Model (HBM; [11–14]), a popular framework for understanding and predicting health behaviors. The model posits people’s actions depend on their perceived susceptibility to and severity of a health threat, perceived benefits of and barriers to a recommended action, plus cues to action and self-efficacy; it is widely used to design and evaluate behavior-change interventions. To test this model in our subject population, we focus on three beliefs: perceived susceptibility to influenza, perceived probability of vaccine side effects, and perceived vaccine effectiveness. We exclude perceived severity of influenza and perceived barriers to vaccination because these dimensions are relatively homogeneous within our study population. Importantly, we aim to elicit students’ beliefs about *their own health risks* rather than their beliefs about the general population, as personal beliefs should be more relevant for their own vaccination decisions. For example, a student’s belief about her own likelihood of contracting influenza should be more informative for her vaccination choice than her belief about the average American’s risk.

We begin by reviewing belief elicitation procedure using the QSR. In a standard QSR task, a participant bets an endowment of $x on the outcomes of an uncertain event. Consider an uncertain event with two possible outcomes, denoted as θ∈{0,1}. Suppose a participant bets a proportion, r, of their endowment on θ=1, and 1−r on θ=0. At the end of the experiment, the participants’ payoff is determined by the realized outcome:


$$\begin{array}{c} \text{Payoff}=\left\{ \begin{array}{c} \text{ x}-\text{x}(\text{1}-\text{r}{)}^{\text{2}},\text{ if }\theta =\text{1},\\ \text{ x}-\text{x}(\text{0}-\text{r}{)}^{\text{2}},\text{ if }\theta =\text{0}. \end{array}\right.\; \end{array}$$ (1)

Suppose a participant believes P(θ=1)=p. A *risk neutral* participant maximizes the expected payoff:


$$\begin{array}{c} \underset{\text{r}}{\text{max}}\;\text{p}\cdot \left(\text{x}-\text{x}(\text{1}-\text{r}{)}^{\text{2}}\right)+(\text{1}-\text{p})\cdot \left(\text{x}-\text{x}(\text{0}-\text{r}{)}^{\text{2}}\right). \end{array}$$ (2)

The optimal solution to this maximization problem is r=p. Thus, the QSR incentive structure in (2) allows us to infer that participants’ unobservable belief p from the observable action r.

A *non-risk-neutral* participant would maximize some form of weighted utility over risky outcomes. Assuming the Rank Dependent Utility model (RDU; [15]), the problem becomes


$$\begin{array}{c} \underset{\text{r}}{\text{max}}\;\omega (\text{p})\cdot \text{u}\left(\text{x}-\text{x}(\text{1}-\text{r}{)}^{\text{2}}\right)+\left(\text{1}-\omega (\text{p})\right)\cdot \text{u}\left(\text{x}-\text{x}(\text{0}-\text{r}{)}^{\text{2}}\right), \end{array}$$ (3)

where ω( ) represents the participant’s cumulative or de-cumulative probability weighting function depending on the rank of the two payoffs, and u( ) represents the Bernoulli utility function. Knowing both functions, we can infer p from r based on the optimal solution of this problem. In practice, auxiliary experimental tasks can be used to identify these functions and recover beliefs [16].

We now consider applying this method to elicit a participant’s belief about their own susceptibility to influenza. In this example, the uncertain event is whether a participant contracts the flu during a typical flu season. Naturally, the procedure requires the participants to bet on their own health outcomes. Note participants cannot know the realized outcome ahead of the bet, so the health outcomes need to be in the *future*. Therefore, we use θ=1 to represent the outcome where they contract the flu in the upcoming flu season, and θ=0 otherwise.

An immediate challenge is that a participant’s health outcome is both unavailable and unverifiable. Unavailability is straightforward: since the health outcome is realized in the future, it will not be available at the end of the experimental session. Consequently, payoffs cannot be determined immediately. A potential solution is to delay payoff until after the season ends, when participants can report back whether they contracted flu. Logistical difficulties aside, this can’t resolve a more fundamental problem: unverifiability. Due to the unverifiable nature of the health outcome, participants can simply bet their entire endowments on θ=0 (I will not get flu) and claim not to have contracted the flu *regardless of their actual experience*. Since the experimenter cannot credibly verify or refute such claim, they have to pay the participant based on their claims. In this case, a participant’s decision to bet all $x on “No” reveals nothing about their underlying belief, rendering the incentivized procedure pointless.

A second challenge is that betting on one’s *own* health outcomes creates opportunities for “hedging.” Risk-averse participants are incentivized to bet more money on adverse health outcomes, such as contracting the flu. This allows them to receive higher monetary payoffs if they contract the flu and compensate for the loss they experience from such an outcome. These losses may include missed hours at work or school, cost of treatment, and utility loss from the pain and suffering. This uncontrolled and unobserved incentive, outside of what the QSR provides, introduces biases into inferences from belief elicitation tasks. To show this, suppose a participant suffers a loss of $L from contracting the influenza virus; then, the problem in (3) becomes


$$\begin{array}{c} \;\underset{\text{r}}{\text{max}}\;\left(\text{1}-\omega (\text{p})\right)\cdot \text{u}\left(\text{x}-\text{x}(\text{1}-\text{r}{)}^{\text{2}}-L\right)+\omega (\text{p})\cdot \text{u}\left(\text{x}-\text{x}(\text{0}-\text{r}{)}^{\text{2}}\right). \end{array}$$ (4)

Here, a risk-averse participant will bet more on θ=1 than they would have without the loss. However, our inference can only be based on the solution for (3), because the loss L is unobservable. Consequently, the inferred belief is biased upward for risk-averse participants who engage in hedging. Conversely, a risk-loving participant will bet less on θ=1 than what they would have, causing a downward bias on the inference of the underlying belief p.

Together, unverifiability and hedging make it infeasible to incentivize elicitation of health beliefs using *participants’ future health outcomes*. As a workaround, we incentivize the procedure using the *past* health outcomes of *others with similar susceptibility to flu.* That is, θ=1 now represents a resemblant peer having contracted flu in the past flu season, and θ=0 the opposite. Essentially, this approach elicits participants’ beliefs about their resemblant peers as proxy beliefs about themselves. First, because the outcome is based on the past flu season, it is now readily available at the end of the experiment session. Second, as the outcome is of others and not one’s own, participants would not know the realized outcome when they place the bets. Third, this eliminates the opportunity for the laboratory participants to manipulate their own reported outcome to ensure maximum payoff of $x. Lastly, this also eliminates potential opportunities for hedging, because the participants simply do not experience losses from others’ adverse health outcomes.

We operationalize this solution using a two-stage design. In the first stage, we conduct a pre-experiment survey and collect past health outcomes from resemblant peers. In our case, the resemblant peers are a randomly selected sample of participants from *the target population* -- undergraduate students at the UMass Amherst. In the second stage, we recruit a different set of participants from the target population to participate in the laboratory experiments. In the experiments, participants bet on whether a random student from the pre-experiment survey had reportedly contracted the flu during the previous flu season. At the end of the experiment, we randomly draw a response from the pre-experiment survey to determine their payoff.

There are a few details worth discussing when implementing this design. First, we use the popular QSR simply as an example to showcase the proposed solution. We do not intend to contribute to the ongoing horse race literature on specific belief elicitation methods. We provide a solution that can be applied to popular incentive compatible elicitation methods. Based on the research topic, context, and desired degree of complexity, researchers can readily change the specific elicitation method in the second stage. To reduce the complexity of the decision task, researchers can use outcome matching [17,18] and probability matching methods [19–21]. To avoid the need to adjust for risk attitudes, researchers can use the Binarized Scoring Rule method [2,22,23].

Second, the purpose of the first stage is to collect the health outcomes of resemblant peers. In this stage, researchers can also replace the pre-experiment survey with other desired data collection methods or sources. For example, if available and accessible, researchers can also use anonymized medical records of resemblant peers from local hospitals and medical centers. These records are arguably more accurate than self-reported outcomes by the peers. As another example, researchers can opt for a post-experiment survey and use the *future* health outcomes of resemblant peers to incentivize the belief elicitation. However, this alternative is logistically more demanding, as it requires extended data collection and delayed payment. Moreover, our objective is to study the causal relationship between health beliefs and vaccination behavior. To do so, we must reveal the actual rate of flu infections *before* the laboratory participants decide whether to take the flu shot or not. As such, we need to have this rate readily available at the end of the experiment session, which was intentionally chosen to be immediately before the beginning of the next flu season.

Third, a critical design choice concerns the definition of “resemblant peers.” Ideally, these peers should share relevant characteristics with laboratory participants. This is to ensure that elicited beliefs closely approximate beliefs about one’s own health. In our setting, undergraduate students at UMass Amherst share many relevant attributes, including age, educational environment, living conditions, local climate, and circulating virus strains. Therefore, the general undergraduate students population is a natural choice as the resemblant peers. Additionally, we explored choosing the students who share even more similarities than the general undergraduate population with each lab participant to further increase the resemblance. Specifically, we also ask our laboratory participants to bet on the health outcomes of a *subset* of undergraduate students who share the same gender, attendance in large-size lectures, dormitory residence status (compared with off-campus housing), and similar hours of sleep. Again, we chose these characteristics because they may affect susceptibility to the flu virus. In Section 5, we discuss this specific design choice in other settings.

Our data analysis shows that the elicited beliefs using this design are highly correlated with the participants’ own health outcomes in the past, suggesting that beliefs about resemblant peers serve as a valid proxy for beliefs about oneself. However, we find no improvement in the relevance of the elicited beliefs based on the matching subgroup. The elicited belief is not significantly different between using the general undergraduate population and using character-matched subgroups. These results are presented in Section 3.

## 2. Literature

The unverifiability issue applies to all *incentive-compatible* elicitation procedures based on scoring rules (such as binarized scoring rule, [22–24]), outcome matching [17,18], and probability matching [19–21]) methods. This is because for these procedures to be incentive-compatible, a verifiable outcome that cannot be manipulated by the participant (often referred to as the “truth” or the “answer key” in the literature) is required to determine participants’ payoffs. As such, our proposed solution can readily supply these verifiable outcomes to these alternative methods.

There are incentive-compatible methods that do not require verifiable income but invoke stringent theoretical assumptions and increased complexity in their implementation. Bayesian Truth Serum (BTS) methods reward respondents for both their own report and for predicting the distribution of others’ reports; theoretically this can make truthful reporting a better strategy even when the true state is unverifiable [25]. In practice, incentive-compatibility and the desirable “surprising-common” scoring rely on informational assumptions (common priors or well-behaved beliefs) and on sufficiently large, well-mixed respondent pools, and the requirement that each subject report a prediction of others’ answers increases cognitive load and reduces transparency [1,25].

Bayesian prediction markets have also been developed to address the unverifiability issue. Peer‑prediction and related multi‑task methods score respondents relative to peers’ reports rather than an external truth, and a large literature has extended the basic peer‑prediction idea to address collusion, heterogeneous workers, endogenous effort, and small populations [26–30]. However, these mechanisms suffer from several practical limitations: many variants require strong informational assumptions (common priors or known signal structures) or many tasks per agent to identify truthful equilibria; multiple equilibria (including uninformative coordinated reports) can exist and are difficult to rule out in the field; collusion-resistant designs or prior‑free variants typically add complexity to payments or require extra tasks/replication, increasing experimental cost and participant burden [26–28].

In contrast, we propose a method that does not require as stringent assumptions for theoretical compatibility as either method. This is because we do not use the health outcomes reported by *the same participants* in the laboratory experiments as the answer key. In doing this, we remove the strategic considerations when subjects provide their report and establish a simple individual decision-making environment. As a result, we do not require the assumption of Bayesian Nash Equilibrium for theoretical compatibility. Our method complements the survey method and the above-mentioned Bayesian methods by striking a balance between task complexity and the desirability of incentive compatibility of the elicitation method.

The hedging issue has been observed in various decision environments and also applies to other elicitation methods. [18,31] show that all incentivized methods fail when participants have a stake in the events of interest. [32] confirm that participants may exploit obvious hedging opportunities. [33] find that many participants hedge in a 2 × 2 coordination game where the opportunity is obvious, and incentives are strong. The hedging issue is also echoed in a concurrent work by [34]. In their paper the utility function is state contingent, rather than state independent. While they provide a solution that solves the heading issue, it cannot simultaneously address the lack of availability and verifiability for health outcomes.

There are also alternative ways of eliciting health beliefs in the literature. Researchers may ask participants to bet on public or official data on health outcomes of interest (e.g., [35]). However, this approach has two disadvantages. First, we may not have the most relevant public or official data for some diseases. In the case of influenza infection, the only official data available are the percentage of influenza infection cases *among people who visit healthcare facilities or are hospitalized* [36]. This percentage reflects several factors other than susceptibility to influenza infection, such as the prevalence of risk factors in the population that lead to more severe cases warranting hospital visits or hospitalization. Second, even when such official data are available, it usually concerns a much broader population than the peer groups used in our design. The participants’ beliefs about the general population may not reflect their beliefs about their own health status. The more reflective the elicited beliefs about their own health status, the more statistical power we may have to detect how and to what extent these beliefs affect vaccination decisions.

A second method often used in health behavior research is surveys or introspective methods to solicit relevant beliefs [37–43]. In these tasks, participants report their health beliefs without incentives for truth telling. In some cases, participants are given a flat payoff. While general evidence is mixed on whether providing incentives to tell the truth improves accuracy, proper incentives can encourage cognitive efforts and enhance the accuracy of elicited beliefs *when the beliefs are inherently difficult to understand and interpret* [44–47]. Regarding health beliefs, [48] examine how well people (1) differentiate and perform simple mathematical operations on risk magnitudes using percentages and proportions, (2) convert percentages into proportions, (3) convert proportions into percentages, and 4) convert probabilities into proportions. They observe that even highly educated participants cannot *directly* formulate numerical probabilities, percentages, and frequencies. In addition, when the beliefs are politically charged in nature, proper incentives can also reduce express reporting [49,50]. This makes the incentivized belief elicitation method more relevant in our context, as vaccination correlates with political ideologies [51,52].

Our approach also relates to the broader literature on proxy beliefs. This general idea has been successfully established and widely utilized in other domains. Just to name a few examples, [53,54] use beliefs about other students (from national data) as proxies to understand own academic expectations. [55] uses a similar application in school/major choice. We apply this well-established idea in the health domain to address issues unique to health data we identified above. Our research design is related to [56]. They performed a meta-analysis of (mis)perceptions of others in field experiments. While most studies included in the meta-analysis focus on comparing the elicited beliefs about others to the truth, i.e., the actual distribution of events among others (section 3.1 pp 430–431), they also correlated the beliefs about others and one’s own characteristics in section 3.5. There they discussed two potential reasons for the widely observed misconceptions: projection bias and motivated reasoning. The “projection bias” due to one’s own experience and opinion is exactly what we hope to observe, which allows us to use the beliefs about others as projected beliefs about self. On the other hand, our use of incentive compatible elicitation procedure can minimize the biases due to motivated reasoning or expressive report [57,58].

There are also several studies that elicited participants’ beliefs about *others’ opinions* in the health domain [59–63]. Some also employed a pre-experiment survey to collect data about opinions of others [59,62]. The beliefs they elicited are used as *a measure of perceived social norm* -- how others’ think about the appropriateness of some health behavior -- *not* as proxies of participants’ own view or opinions*.* As such, the general theme of these studies is to examine how the *(mis)perceived opinions of others* affect one’s health behavior, while our goal is to see how *perceived health risks of one’s own* is *proxied by one’s beliefs about others,* affect health behavior. We establish the elicitation method here and explore the behavioral consequences in a separate paper. In this aspect, our approach is more similar to the practices outside of the health domain, exemplified by [53–55]. Also importantly, none of these studies used incentive compatible elicitation method or identified the issues in its application. As we discussed above, incentive compatible measures are helpful in promoting truth telling and minimizing the bias due to “motivated reasoning.” [57] finds that beliefs about others’ behavior are biased and self-serving (expressive) when elicited without incentives. Biases are reduced when using an incentivized elicitation method that ties monetary outcomes to accuracy and makes those outcomes salient. The lack of incentive for truth telling could be especially problematic considering some of the aforementioned studies look at highly stigmatized health issues such as HIV infections.

In summary, this study contributes to the literature on belief elicitation methodologies. It identifies and resolves the challenges of availability, verifiability, and hedging when eliciting one’s own health beliefs using popular incentive compatible procedures. It may improve upon the standard, unincentivized survey method by reducing motivated reasoning or expressive reporting caused by political bias and improving cognitive effort and accuracy. Moreover, our proposed method complements recent incentive-compatible elicitation based on publicly available health statistics. It enhances the relevance of elicited beliefs using a more relevant population in the elicitation procedure.

## 3. Experiment procedures

The experimental design consists of two major stages: the pre-experiment survey stage and the laboratory stage. In the pre-experiment survey, we collect the health outcomes from a random sample of college students. In the laboratory stage, we use the outcomes of the pre-experiment survey to incentivize belief elicitation procedures. The pre-experiment survey respondents are considered the resemblant peers of our laboratory participants. Additionally, we elicited participants’ risk attitudes to correct for the biases of risk attitudes in the inference of beliefs.

### 3.1. Pre-experiment Survey

Before laboratory sessions, we conducted an anonymous survey with Amherst undergraduate students at UMass Amherst. During the survey, we collected data on students’ health outcomes during the past flu season, including whether they (1) contracted the flu, (2) received the flu shot, (3) experienced any side effects after taking the flu shot, and (4) contracted the flu despite receiving the flu shot. We approached the survey respondents randomly at a university café.

Flu season occurs in the fall and winter (October to May) and peaks between December and February [36]. The survey was conducted between April 17 and 27 in 2018, toward the end of the 2017–2018 flu season so the respondents could easily recall the health outcomes. We chose this timing to avoid potential misreports owing to the difficulty in recalling if the survey was to be conducted long after the flu season. The survey had 9–11 questions, depending on the participant’s response (see S3 Appendix for the complete list of questions). The first two questions screened the respondents to ensure whether they were UMass students and at least 18 years old. If a respondent passed the initial screening, the following five questions were regarding their gender, exercise frequency, sleep duration, whether they attended large enrollment courses, and whether they lived in the University dormitory. We selected these characteristics because they could potentially affect the risk of influenza infection. It allowed us to elicit participants’ beliefs about the subgroup of survey respondents with the same characteristics at the laboratory stage.

Questions 8–11 were closed-ended questions about the abovementioned flu-related facts. According to the official CDC Influenza Vaccine Information Statements [64], we specified flu infection symptoms and potential side effects, thus reducing misreports due to a common misunderstanding of what counts as flu and its side effects. Moreover, we asked questions on side effects and vaccine ineffectiveness only after confirmation from the respondents regarding receiving a flu vaccine.

### 3.2. Laboratory stage

For the laboratory sessions, the participants were recruited using the standard ORSEE online recruitment system [65] between September 11 and October 30, 2018. Each participant sequentially performed risk preference tasks, incentivized belief elicitation tasks, time preference tasks, and a post-experiment survey. The experiment is programmed in Ztree [66]. Time preference tasks are not analyzed in this study. As participants always complete risk and belief tasks first without knowing what is coming next, the presence of time preference tasks should not affect the results discussed in this study.

### 3.3. Risk preference

Controlling for risk preference is essential to ensure an unbiased inference of participants’ beliefs from their decisions in belief elicitation tasks. [67] and [16] establish empirical evidence suggesting that risk aversion biases participants’ bets on belief elicitation tasks by 50%. To correct this bias, we included separate tasks to elicit the participants’ risk preferences, following [16]. Alternatively, one can use the risk adjustment proposed in [68]. We elicited the participants’ risk preferences by asking them to make 40 binary lottery choices. The participants were presented with 40 pairs of lotteries, one pair at a time. Each lottery offers a different chance of winning various monetary prizes. The participants selected the one they preferred to play for each pair of lotteries. Forty pairs of lotteries were carefully chosen based on the design proposed by [69] to ensure a robust inference of participants’ risk preferences. Table B.1 lists the probabilities and prizes of the 40 lotteries used in this paper. We also have 20 other lottery choices that involve losses. However, these choices are not used because the belief elicitation tasks do not include the loss domain in our estimation model. Including the last 20 lottery choices introduces unnecessary noise in identifying the Bernoulli utility function and probability weighting functions in the gain domain as we need to further assume whether participants fully integrate the additionally provided endowment with losses.

To facilitate the participants’ understanding, each pair of lotteries was presented to the participants visually using two pie charts (see S1 Appendix, Figure A1). The pie is divided into colored areas proportional to the probability of winning each prize. Once a participant submits their choice, the selected lottery remains on the screen and is played out to determine their payoff in the current task. A white needle then appears at the top of the pie. The pie starts spinning and stops randomly. The participant wins the prize for the colored area where the white needle rests. Subsequently, the next lottery pair appears on the screen. To control for the order effect, the order of the lottery pairs and positions of the two pies are randomized for each participant. After a participant completes all tasks, we randomly choose one task, and the prize that a participant wins in that task is their payoff for this part of the experiment.

#### 3.3.1. Belief elicitation.

In this part of the experiment, we elicited participants’ health beliefs through eight betting tasks. These beliefs pertain to the chances of (1) flu infection, (2) others receiving influenza vaccination, (3) experiencing any vaccine side effects, and (4) flu infection after receiving an influenza vaccination. The four flu-related questions in the pre-experiment survey matched the beliefs that we wanted to elicit, allowing us to use the pre-experiment survey responses to incentivize decisions in the current tasks. To simplify the description below, we use the chance of flu infection as an example to explain the procedure.

To elicit a participant’s belief on the chance of flu infection, we ask each participant to bet whether a randomly selected response in the pre-experiment survey is “Yes” or “No” for the flu infection question. The participant places their bet by allocating 100 tokens between the two possible outcomes: “yes” or “No.” The token allocation determines the payoffs the participant receives when the randomly selected response from the pre-experiment survey turns out to be “Yes” or “No.” The specific payoff function is as follows:


$$\begin{array}{c} \text{Payoff}=\left\{ \begin{array}{c} @l\$\text{20}-\$\text{20}\times (\text{1}-\text{r}{)}^{\text{2}}\;,\text{ if the randomly selected response is ``Yes"}\\ \$\text{20}-\$\text{20}\times (\text{0}-\text{r}{)}^{\text{2}},\text{ if the randomly selected response is ``No"} \end{array}\right.\; \end{array}$$

where r represents the proportion of tokens allocated to “Yes.”

To maximize the expected payoff, a risk-neutral participant should allocate their tokens so that the percentage of tokens allocated to “Yes” equals their subjective belief about the percentage of “Yes” responses in the pre-experiment survey. That is, a participant’s token allocation corresponds to their belief in the chance of a flu infection. For example, if a risk-neutral participant places 30 tokens to “Yes,” we infer that their subjective belief regarding the probability of contracting flu on campus is 30%. Risk-neutral assumptions are not a critical limitation of QSR. One can combine the choices in the binary lottery tasks in Section 3.2 to recover beliefs under much more flexible utility assumptions [16]. We adopted a rank-dependent utility framework throughout our data analysis and jointly estimated the participants’ beliefs and utility functions. S2 Appendix provides the details of this estimation procedure.

To reduce cognitive effort and promote the participants’ understanding, we visually presented payoffs to each participant using the interface suggested by [16]. The participants use a slider to allocate tokens and instantly observe the calculated QSR payoffs for the two possible outcomes. The payoffs are displayed in the intuitive bar chart (see Appendix A, Figure A2). Once satisfied with a particular allocation, they submit it as their final bet. The instructions were given using a practice question, during which the participants were encouraged to familiarize themselves with the interface and try different token allocations to determine the corresponding payoffs. Before asking the participants to make a bet, we presented each question using the exact wording as asked in the pre-experiment survey. We also explain the timing of the pre-experiment survey and how it could help the survey respondents to better recall their experiences in the past flu season. Thes instructional procedures reduce noise in the participants’ beliefs owing to their uncertainties about our survey method.

Using the same method, we subsequently asked the participants to bet on their responses to the other three questions in the pre-experiment survey, thus eliciting their health beliefs about the influenza vaccination rate, chances of side effects, and chances of influenza infection despite receiving vaccination.

To examine the extent to which similarities between the laboratory participants and pre-experiment survey respondents affected one’s bet on beliefs, we further narrowed down the survey respondent group to a subset that shared similar lifestyle characteristics with each laboratory participant. We implemented this by asking laboratory participants to participate in a short survey on four additional characteristics: gender, whether they attended large enrollment courses, slept at least seven hours at night on average, and lived in the university dormitory. After the laboratory participants provided their answers, we informed them how many respondents in the pre-experiment survey provided answers that matched their answers (see S1 Appendix, Figure A3). The laboratory participants were then instructed to place an identical set of four flu-related bets; however, we now determined their payoffs using *only* a random response drawn from those of their respective matching subgroups. We made the differences between the first and second four bets as salient as possible. In addition to the verbal instructions, we highlighted this feature in red text on the betting interface (see S1 Appendix, Figure A4).

After the participants placed all eight bets, we randomly selected one bet to determine their payment for this part of the experiment. For the chosen bet, we then asked each participant to draw a random response (either “Yes” or “No”) to the selected question in the pre-experiment survey (see S1 Appendix, Figures A5 and A6). Participants were paid based on their bet and the outcome of the random response. They were informed regarding the randomly drawn response, their payments, and the actual percentage of “Yes” responses to the selected question in the pre-experimental survey. This feedback step concludes the belief elicitation procedure.

Both data collection procedures were approved by the Institutional Review Board of University of Massachusetts Amherst (Protocol ID: 2018–4670 and 2018–4690).

#### 3.3.2. Participants’ own health outcomes.

Following the belief elicitation tasks, participants filled out a questionnaire about whether they, in the past flu season, contracted the flu, received a flu shot, and experienced any side effects or ineffectiveness of the flu shot (see S4 Appendix for details). The health outcomes collected here allow us to examine whether the elicited belief regarding one’s peers in Section (3.3.1) is correlated with one’s own past health history reported in the current questionnaire. If a correlation exists, we can treat it as supporting evidence that participants’ beliefs about their peers reflect their beliefs about their own health outcomes. Moreover, we can compare the strength of such a correlation when the peer group comprises students from the same university or a subset of students who share similar lifestyle characteristics with the laboratory participants. If a stronger correlation is detected when the subset of students is used as peers, we can support the notion that the more similar the peer group is to oneself, the better the calibration for one’s health belief using our proposed method. To ensure a consistent interpretation of what describes a flu infection and its side effects, we used identical wording in this questionnaire compared with those used in the pre-experiment survey.

One may be concerned with the issues of accuracy in the elicited beliefs and reported health outcomes due to the lapse of time between the previous flu season and the time we conduct the laboratory experiments. First, the timing of the laboratory sessions is a design feature to capture the real-world decision-making process. We intentionally choose to conduct the lab experiment sessions close to the beginning of the new flu season because this is typically when people decide whether to take a flu shot or not. In this decision process, one would recall what happened in the past season, think about what would likely happen in the upcoming season, then decide on whether to take the shot. Second, note there are two items that we elicited from the lab participants: their beliefs and their own past health outcomes. These two items are related but distinct. [70,71] show that recollection of beliefs and past events are separate factors, and changes in one (e.g., fading vividness of an event) don’t necessarily entail changes in the other (the belief might persist). In addition, studies such as [70–72] show that beliefs are more stable and less prone to decay over time than recollection of events. Therefore, the recall of beliefs as outlined in Section 3.3.1 is less prone to the accuracy concern from the passage of time. The recall of health outcomes outlined in Section 3.3.2 can indeed be nosier due to less perfect recall. However, the effect would be a reduction of the statistical power of detecting the correlation between one’s beliefs and their own past outcomes.

### 3.4. Empirical strategy

Next, we evaluate the effectiveness of our belief elicitation procedure, that is, whether the elicited beliefs genuinely reflect what the participants truly believe about their own health. The difficulty in evaluating the performance of a belief elicitation procedure is that *a true subjective belief* is unobservable; therefore, it cannot be used as a benchmark for comparison. We choose the participants’ own flu facts in the past flu season as benchmarks to show the relevance between the elicited beliefs and participants’ beliefs about their own health.

To understand why we chose participants’ own flu facts as the benchmarks, it is important to review the alternative approaches in the literature reviewed in [2] and explain why they do not work in our case. First, some studies examine whether the elicited beliefs are consistent with the objective probabilities of random devices that are used to induce these beliefs (e.g., [22,67,73–75]). We cannot adopt this approach because the selected beliefs are not induced.

Second, some studies compare the elicited beliefs with the empirical distribution of the underlying random events. For example, some studies elicit participants’ beliefs about other players’ behavior in a game and then compare whether the elicited beliefs are consistent with the observed behavior of other players (e.g., [44,76,77]). This approach is problematic because there is no reason to expect the participants’ beliefs to correspond to an empirical distribution. In our case, deviations in the participants’ beliefs from their actual chances are expected. These deviations are of primary interest to researchers in this field. Specifically, we are interested to see if participants significantly underestimate the probability of flu infection on college campuses.

Third, some studies use the degree to which elicited beliefs are consistent with *related actions* as a benchmark for evaluation (e.g., [78–81]). This approach is essentially a *joint* test of the accuracy of the elicited beliefs *and* decision model linking beliefs and actions. It is *not* a proper benchmark when the decision model itself is up for testing, as in our study, we elicit participants’ beliefs *to test* the Health Belief Model (HBM). In fact, in a separate paper [82], we test the HBM by evaluating the effects of random shocks on the elicited beliefs and observing the subsequent behavioral changes, where we established supporting evidence regarding the HBM using the beliefs elicited with the method proposed in the current paper.

Lastly, one can compare the elicited beliefs across different procedures, that is, we can use elicited beliefs from another procedure as benchmarks (e.g., [23,47]). This type of evaluations can only show that at least one procedure is mismeasured at best, without elucidating which procedure is the better one.

**Fig 1:**
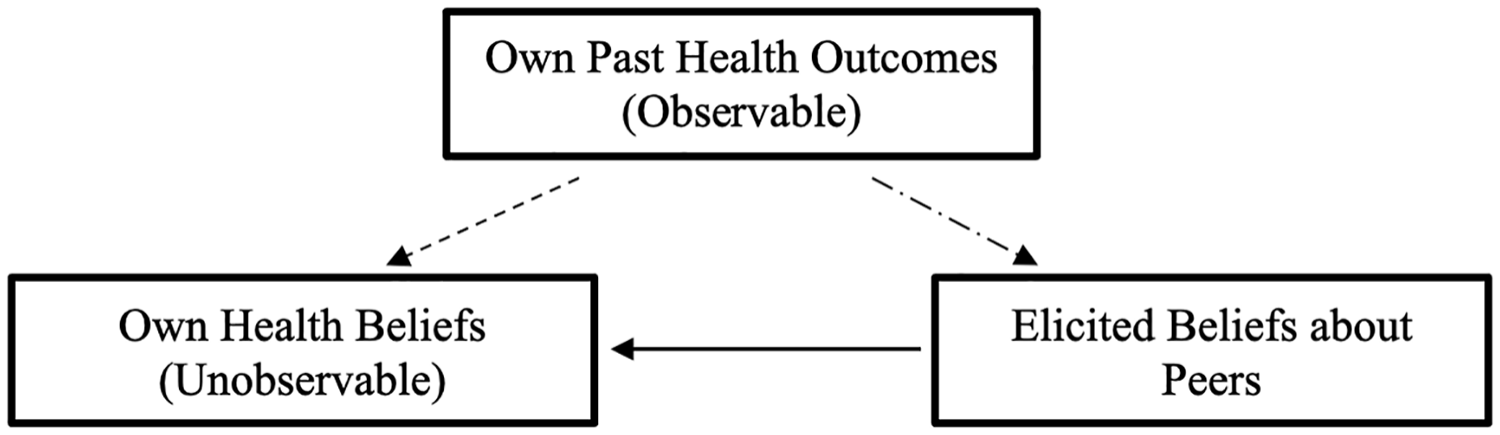
Benchmarks to evaluate the relevance of the elicited beliefs.

Now, we explain the logic behind our choice of benchmarks. Recall that our goal is to elicit one’s health beliefs about their *own* susceptibility to influenza virus and their *own* chance of experiencing the side effects or ineffectiveness of the influenza vaccine (bottom left box, Fig 1), which we cannot directly elicit for reasons discussed previously. We resort to eliciting their beliefs about their close peers and show that the elicited beliefs about peers are reflective of the participants’ beliefs about their own health (solid arrow, Fig 1). To achieve this, we used observable past health outcomes (top box, Fig 1) as a proxy for one’s own health beliefs. The logic is that one’s own (unobservable) health belief is determined by one’s own past health outcome (dashed arrow, Fig 1), observing a correlation between the elicited beliefs and participant’s past health outcome (dash-dotted arrow, Fig 1) serves as a strong indicator that the elicited belief reflects one’s health belief regarding themselves.

The idea that past experiences inform future beliefs is corroborated in both psychology and economics. For example, [83]establishes that the human brain uses the same neural network to remember the past and imagine the future. [84] introduces the concept of a “response shift,” where people adapt their internal standards after a health crisis. They show that “turning point” (like a stroke or cancer diagnosis) changes an individual’s subjective health beliefs. The health events used in this study could also in theory lead to behavior and lifestyle changes, yet they are still found to affect future beliefs. Using data from 11 countries, [85] provides empirical evidence that personal health history is a primary driver of future-oriented financial planning. Individuals who personally experienced a negative health event were 25% more likely to intend to purchase income protection insurance.

Based on this strategy, we survey the laboratory participants’ own health outcomes in the past flu season and check whether they correlate with the elicited beliefs in the right direction. We run regressions for each elicited belief in the *relevant* health outcomes of each participant. Using the chance of influenza infection as an example, we expect that the elicited beliefs about influenza infection are higher for participants who contracted the flu in the past flu season. Similarly, we may observe that participants’ past experiences of the side effects/ineffectiveness of the influenza vaccine have similar effects on the corresponding beliefs elicited using our procedure. Moreover, we check whether a participant’s flu shot decision affects their beliefs about others’ flu shot decisions. To correct risk attitudes, we include the participants’ binary lottery choices and jointly estimate the effects of one’s past health outcomes on the elicited beliefs with the participants’ risk preferences using the established procedure by [16]. We provide the detailed estimation procedures in S2 Appendix. To constraint the estimates of individual beliefs to be between 0 and 1, we use the following transformation to explore the correlation between the estimated beliefs and the relevant past experiences:


$$\begin{array}{c} \text{ln}\left(\frac{P_{i}}{\text{1}-P_{i}}\right)=\beta_{i\text{0}}+\beta_{i1}\cdot X_{is}, \end{array}$$ (5)

where s is the index for a participant and $X_{is}$ represents the relevant past experiences and $P_{i}$ is the beliefs that need to be estimated based on participants’ reports.

## 4. Results

We conduct the experiments at the Cleve E. Willis Experimental Economics Laboratory at the UMass Amherst. We elicited eight beliefs from each of the 254 participants we recruited for the experiment. The average duration of the experiment is two hours, with an average payoff of $39. Table 1 provides the demographic composition of the participants. Based on the estimation procedures in S2 Appendix, we report our main results in Table 2. We report the linear coefficients specified in equation (5), $\beta_{i\text{0}}\text{ and }\beta_{i1}$, under “Model 1” of Table 2. For ease of interpretation, we also include the implied marginal effect on the relevant beliefs under the column of “Model 1 ME.” We will use these figures as basis for discussions. Recall that we elicited participants’ beliefs about both the general undergraduate student population (population) and the subgroup of students who shared similar characteristics (subgroup). For ease of comparison, we present the results for the same belief side by side, with the results based on the general population in the left panel and the results based on the subgroup in the right panel. For succinctness, we report belief-related parameters here and the estimates of nuisance parameters in Appendix Table B.2.

**Table 1:** Summary Statistics of Participants’ Demographics.

Variable	Number of Participants
**Age**	
18	70
19	79
20	51
21	33
22 and above	21
**Sex**	
Female	141
Male	113
**Race**	
White	150
African American	13
Asian/Pacific Islander	65
Hispanic or Latino	6
Other	20
**GPA**	
Below 2.0	2
2.0-2.99	24
3.0-3.99	74
3.5-4.3	154
**Taken classes in**	
Economics	132
Statistics	162
**Smoker**	32
**Athlete**	45

**Table 2:** Correlation between participants’ beliefs and relevant health status.

Elicited Belief	Own Health Outcome / Characteristics	Population		Subgroup	
		Model 1	Model 1 ME	Model 1	Model 1 ME
P Flu	Flu2017	1.025***	0.245***	1.147***	0.272***
		(0.207)	(0.050)	(0.248)	(0.060)
	Constant	−0.820***	0.306***	−0.907***	0.288***
		(0.0891)	(0.019)	(0.0983)	(0.020)
P Flu Shot	Flushot2017	0.643***	0.147***	0.786***	0.180***
		(0.152)	(0.034)	(0.166)	(0.036)
	Constant	0.264**	0.566***	0.167	0.542***
		(0.0997)	(0.024)	(0.100)	(0.025)
P Side Effect	No side effect after flu shot	−0.927***	−0.223***	−0.941***	−0.221***
		(0.189)	(0.044)	(0.232)	(0.051)
	Side effect after flu shot	1.390***	0.283***	0.755***	0.179***
		(0.267)	(0.045)	(0.228)	(0.052)
	Constant	0.183	0.546***	0.0298	0.507***
		(0.122)	(0.030)	(0.135)	(0.033)
P Flu After Shot	No flu after flu shot	−0.898***	−0.110***	−0.656*	−0.092**
		(0.309)	(0.031)	(0.263)	(0.033)
	Flu after flu shot	0.308	0.055	0.364	0.069
		(0.660)	(0.127)	(0.634)	(0.130)
	Constant	−1.352***	0.206***	−1.264***	0.220***
		(0.134)	(0.022)	(0.132)	(0.023)
Observations		12,192	12,192	12,192	12,192

* p < .05 ** p < .01 *** p < .001

First focusing on the “Population” panel of Table 2, these provides the elicited beliefs when we use the general undergraduate population as the resemblant peers. Consistent with our expectations, those who contracted the flu in the previous flu season (Flu2017 = 1) believe in a higher risk of influenza infection (P Flu), resulting in a 24.5% increase in the subjective probability of contracting the flu. Furthermore, those who receive a flu shot (Flushot2017 = 1) have a 14.7% higher subjective probability about how likely others are to receive the flu shot (P Flu Shot). For side effects and ineffectiveness, we include an interaction term between participants’ flu shot decisions and their own experience, while using participants who do not take the flu shot as the baseline group for comparison. Compared with participants who do not take the flu shot, subjective beliefs about side effects (P Side Effect) are 22.3% lower for those who do not experience side effects after taking the flu shot (no side effects after flu shot = 0) and 28.3% higher for those who do (side effects after flu shot = 1). Concerning participants’ beliefs about ineffectiveness (P Flu After Shot), we find a significant reduction of 11% when participants do not experience ineffectiveness (No Flu After Shot = 1).

The above findings hold if we look at the elicited beliefs based on the matching subgroup, with only small and insignificant differences in the scale. Using the beliefs of contracting the flu as an example, the elicited beliefs based on general population are 30.6% among those who did not contract flu, versus 28.8% based on the matching subgroup. The beliefs are 24.5% higher for those who contracted flu if we use the population-based beliefs, versus 27.2% higher if we use the matching group-based beliefs. The same observations hold true for the probability of taking flu shot, experiencing side effects and ineffectiveness after taking flu shot. These findings indicate that when it comes to eliciting flu related health beliefs of undergraduate students, we can simply use the general undergraduate population as the resemblant peers. Further narrowing it down to matching subgroups does not improve the relevance of the elicited beliefs.

Model 1 includes relevant experiences for each belief as independent variables. It shows that participants factor in their own relevant health outcomes and project their beliefs about themselves when they form beliefs about their peers. Therefore, the elicited beliefs about peers reflect their own health beliefs. As a falsification test, we show no correlation between beliefs and irrelevant health experiences. For instance, participants’ experiences of side effects should not affect their beliefs about the possibility of flu infection. We report this as Model 2 in Table 3 and include Model 1 in the table for easy comparison. Starting with the chance of influenza infection (P Flu), consistent with our predictions, we do not find this belief affected by the participants’ experiences of side effects or ineffectiveness. We draw similar conclusions from elicited beliefs about how likely a participant’s peers are to receive a flu shot. The chance of side effects (P Side Effect), again consistent with our expectation, holding constant experiences of side effects, is not affected by experiences of ineffectiveness, which is an irrelevant experience for this belief. We show this by further including the experience of ineffectiveness (FluAfterShot2017) into the interaction between Flushot2017 and SideEffect2017. We then perform Wald tests on the null hypotheses that the coefficients are similar for the pair of interaction terms that take the same values in Flushot2017 and SideEffect2017 but different in FluAfterShot2017. In most tests, we cannot reject the null hypothesis. A few exceptions do exists, as we reject the null hypothesis that the coefficients between 1.Flushot2017 # 1.SideEffect2017 # 0.FluAfterShot2017 and 1.Flushot2017 # 1.SideEffect2017 # 1.FluAfterShot2017 are the same at the 1% significance level.

**Table 3:** Correlation between participants’ beliefs and *irrelevant* health status.

Elicited Belief	Own Health Outcome	Population		Subgroup		
		Model 1	Model 2	Model 1		Model 2
P Flu	Flu2017	1.025***	0.812**	1.147***	0.864**	
		(0.207)	(0.264)	(0.248)	(0.309)	
	Flushot2017		−0.470**		−0.496*	
			(0.180)		(0.297)	
	SideEffect2017		0.0285		−0.122	
			(0.273)		(0.297)	
	FluAfterShot2017		1.025		1.664	
			(0.745)		(1.083)	
	Constant	−0.820***	−0.624***	−0.907***	−0.682***	
		(0.0891)	(0.103)	(0.0983)	(0.110)	
P Flu Shot	Flu2017		−0.405		−0.149	
			(0.223)		(0.266)	
	Flushot2017	0.643***	0.505**	0.786***	0.577**	
		(0.152)	(0.172)	(0.166)	(0.192)	
	SideEffect2017		0.211		0.497	
			(0.241)		(0.258)	
	FluAfterShot2017		0.666		0.584	
			(0.527)		(0.808)	
	Constant	0.264***	0.322**	0.167	0.188	
		(0.0997)	(0.108)	(0.100)	(0.105)	
P Side Effect	Flu2017		0.599		0.299	
			(0.340)		(0.366)	
	1.Flushot2017 # 0.SideEffect2017 # 0.FluAfterShot2017		−0.783***		−0.819***	
		−0.927***	(0.179)	−0.941***	(0.225)	
	1.Flushot2017 # 0.SideEffect2017 # 1.FluAfterShot2017	(0.189)	−2.726*	(0.232)	−3.168	
			(1.225)		(2.527)	
	1.Flushot2017 # 1.SideEffect2017 # 0.FluAfterShot2017		1.552***		0.724**	
		1.390***	(0.290)	0.755***	(0.227)	
	1.Flushot2017 # 1.SideEffect2017 # 1.FluAfterShot2017	(0.267)	−0.0193	(0.228)	1.066	
			(0.480)		(0.824)	
	Constant	0.183	0.101	0.0298	−0.0108	
		(0.122)	(0.122)	(0.135)	(0.139)	
P Flu After Shot	Flu2017		0.458		0.270	
			(0.455)		(0.301)	
	1.Flushot2017 # 0.FluAfterShot2017 # 0.SideEffect2017		−1.143*		−0.563	
		−0.898***	(0.455)	−0.656*	(0.319)	
	1.Flushot2017 # 0.FluAfterShot2017 # 1.SideEffect2017	(0.309)	−0.277	(0.263)	−0.694	
			(0.319)		(0.354)	
	1.Flushot2017 # 1.FluAfterShot2017 # 0.SideEffect2017		−0.872		−0.0954	
		0.308	(1.068)	0.364	(0.876)	
	1.Flushot2017 # 1.FluAfterShot2017 # 1.SideEffect2017	(0.660)	1.175***	(0.634)	0.682	
			(0.265)		(0.679)	
	Constant	−1.352***	−1.421***	−1.264***	−1.301***	
		(0.134)	(0.154)	(0.132)	(0.144)	
Observations		12,192	12,192	12,192	12,192	

* p < .05 ** p < .01 *** p < .001

Similarly, holding constant the experiences of whether a participant contracts flu after vaccination, the elicited beliefs about the chance of ineffectiveness (P Flu After Shot) remain mostly unchanged when we further introduce irrelevant experiences of side effects into the regression. We performed Wald tests similar to what we did earlier for P Side Effect. We include the experience of side effects (SideEffect2017) into the interaction between Flushot2017 and FluAfterShot2017. We then perform Wald tests on the null hypotheses that the coefficients are similar for the pair of interaction terms that take the same values in Flushot2017 and FluAfterShot2017 but different in SideEffect2017. We find that the experience of side effects affected participants’ beliefs about ineffectiveness in the general population. This finding could be explained by the general confusion between the symptoms of side effects and influenza infection upon receiving a flu shot.

To summarize, we find that elicited beliefs are correlated with participants’ own relevant experiences and are generally not correlated with irrelevant experiences. In addition, the direction of the correlation with relevant experiences is consistent with the hypothesis that they substitute their own experiences in forming beliefs about their peers. Considering all evidence, we conclude that elicited beliefs regarding peers are good indicators of participants’ beliefs about their own health status.

## 5. Discussion

In this section we define the concept of “resemblant peers” and discuss how such peers can be selected through some examples. Although it is impossible to provide an exhaustive list of who constitutes “resemblant peers” for all health-related beliefs, our goal is to offer practical guidance on how to apply our method across different contexts.

Researchers should begin by identifying the individual characteristics most relevant to the health outcome of interest, ideally drawing on medical research, clinical guidelines and established consensus. These characteristics should include *external factors*, such as the quality of medical care (e.g., developing vs developed countries, urban vs rural settings), living environment, or occupational exposures. They should also include *internal factors*, such as age, gender, comorbidities, risk factors, genetic markers, family medical history, and other related health behavior (smoking or drug use). “Resemblant peers” can then be defined as individuals who share these relevant characteristics with the participant.

At the same time, researchers must balance the degree of resemblance with the quality and feasibility of data that can be obtained from the peers. Because the beliefs are incentivized based on these data, poor data quality can undermine participants’ trust and reduce the reliability of elicited beliefs. If strict resemblance criteria reduce the peers to a very small or hard-to-reach population, it may even be infeasible to collect sufficient data to incentivize the belief elicitation procedure. In such cases, researchers may consider relaxing the resemblance requirement along characteristics that are relatively less important or particularly restrictive.

This consideration is especially relevant for individuals who belong to the minority group with respect to some characteristics. In the extreme case in which an individual is uniquely defined by the relevant characteristics, surveying “peers” would effectively amount to surveying the individual themselves. Taking our application as an example, we considered four additional characteristics relevant to susceptibility to influenza infection to maximize resemblance: gender, attendance at large classes, adequacy of sleep, and residence in a dormitory. Although the pre-experiment survey includes 438 respondents in total, subgroup sizes defined by these additional characters range from 10 to 121 individuals. For the laboratory participants who belong to the smallest subgroups, it may be preferable to relax one matching criterion to expand the peer group and improve the quality of the elicited belief measures. Below we detail two potential use cases on how to identify “resemblant peers”.

*Example 1: Eliciting pregnant women’s beliefs about preeclampsia*

Preeclampsia is a serious pregnancy‑associated condition marked by high blood pressure and signs of organ dysfunction after 20 weeks’ gestation. Low-dose aspirin can be used to prevent preeclampsia and related morbidity and mortality. To study the accuracy of pregnant individuals’ beliefs about their own risk—and how these beliefs affect uptake of preventive treatment—it is necessary to elicit beliefs about individual risk. However, the risk varies substantially across individuals based on multiple risk factors, making elicitation based on population-level statistics potentially misleading.

According to the practice advisory of American College of Obstetricians and Gynecologist [86], primary risk factors include: history of preeclampsia, multifetal gestation, chronic hypertension, pregestational type 1 or 2 diabetes, kidney disease and autoimmune disease. Moderate risk factors include nulliparity, obesity, family history of preeclampsia, black race (as a proxy for underlying racism), lower income and older age (35 years or older), personal history factors, and in vitro fertilization. In addition, geographical locations also affect the likelihood through many underlying factors such as healthcare quality and access, socioeconomic status, environmental exposures [87,88].

Given these findings, the resemblant peers for a pregnant individual for the purpose of eliciting *this* belief would be the pregnant women who live in the same geographic area and have all the risk factors that she has. Unlike influenza infection, most pregnant individuals receive care from obstetricians or midwives. As such, researchers can replace the pre-experiment surveys with anonymized electronic medical records from the local health-care providers, where access is available. However, for pregnant people with rare risk factors -- such as autoimmune disease -- researchers might need to trade off the most perfect matching of risk factors against the availability of matching cases in the medical records. To implement the QSR, researchers can ask participants to bet on whether a randomly selected pregnant individual similar to themselves would end up getting diagnosed of preeclampsia. This way we can elicit a participant’s belief about their own chance using her proxy belief on the resemblant peers.

*Example 2: Belief elicitation for genetic disorders*

Consider a setting in which researchers wish to elicit an individual’s belief on their likelihood of having a genetic disorder and study how this belief influences decision to undergo testing or treatment. Or in a similar vein, researchers may be interested in parents’ belief about whether their newborn has a genetic disorder and how these beliefs affect their decision on whether to subject the child to potentially invasive testing.

Factors affecting the likelihood of genetic disorder include ethnicity (e.g., sickle cell trait in regions with malaria history), consanguinity (e.g., marriage between close relatives), family history and inheritance patterns (e.g., whether parents are carriers), advanced maternal or paternal age at conception, maternal exposure to teratogens, infections, or toxins during pregnancy, and maternal lifestyle factors (e.g., smoking, alcohol use or poor nutrition during pregnancy).

Based on these medical findings, the resemblant peers should, at the minimum, share ethnicity and geographical region. In addition, if an individual has information about their family history, peers can be further matched on these dimensions. For example, if an individual knows that one parent has the genetic disorder, the resemblant peers should be further restricted to those who also have an affected parent. In the context of the newborn testing, if the mother smoked during pregnancy or was of advanced maternal age, the peers should newborns whose mothers share these characteristics. Researchers can then survey these peers about their testing outcomes or intervention effectiveness, or ideally, draw on administrative or clinical records from testing agencies. Researchers can then ask an individual to bet on whether a randomly selected person with matching characteristics tested positive or negative, or treated successfully or not, for the suspected genetic condition.

## 6. Conclusion

Recognizing the practical challenges of using Quadratic Scoring Rule to elicit one’s own health beliefs, we propose a practical and simple solution: using health beliefs about highly resemblant peers as proxies for health beliefs about oneself. We established the validity of the proposed procedure in the context of the HBM and influenza vaccination decisions. While our target is participants’ health beliefs about their own susceptibility to the influenza virus, chances of experiencing side effects, or vaccination ineffectiveness, we cannot ask participants to bet directly on their own health outcomes because of concerns over the lack of outcome availability, inability to verify self-reported outcomes, and hedging. All these concerns lead to a loss of control in the incentive structure that rewards participants’ truthful revelations of their underlying beliefs, losing the benefits of using incentivized procedures to elicit beliefs. To address these issues, we used the health outcomes of peers to incentivize the procedure. Thus, we can elicit participants’ beliefs about those with similar health status and living environment. Compared with eliciting participants’ beliefs about a more general and unrelatable population, we consider their beliefs about the “resemblant peers” as the best proxy for their health beliefs.

While we showcase this solution using the popular QSR method, the challenges are common among other popular elicitation methods, such as outcome and probability matching methods and the Binarized Scoring Rule. Researchers can freely combine other popular elicitation methods suitable to their subject pool using the first stage of the experiment, the pre-experiment survey among peers, as the answer key or truth. In addition, although the procedure is developed and tested in the context of influenza vaccination, it can be easily adapted to other topics that face similar challenges. A pre-experiment survey is a simple and powerful tool for acquiring otherwise unavailable outcomes required in belief elicitation procedures. One example is eliciting participants’ beliefs about environmental or political events that will occur in the distant future. Researchers can elicit participants’ beliefs about their like-minded peers’ beliefs to analyze their own beliefs.

Researchers can also adjust the proposed method based on the purpose of belief elicitation. For the current paper, the timing of the experiments is ultimately guided by the goal to study the causal mechanism between these beliefs and flu shot decisions. As such, we intentionally choose to conduct the lab experiment sessions close to the beginning of the new flu season, a typical time when people decide whether to take a flu shot or not. In this decision process, one would recall what happened in the past season, think about what would likely happen in the upcoming season, then decide on whether to take the shot. While we consider this choice a strength in achieving our goal, we can see why researchers would alternatively want to conduct the lab experiment right after the pre-experiment survey for better recall of beliefs and events among the laboratory participants. It would be an interesting extension to compare the differences in beliefs between the two timings, as well as how strongly the new information affects later decisions between the two time points.

As noted in [1], there is a trade off between theoretical incentive compatibility, which promotes truth telling, and implementation complexity, which demands subjects’ understanding and mathematical sophistication. On the spectrum of this tradeoff, the survey method is least complex but lacks incentive compatibility; some methods add modest complexity to achieve incentive compatibility but require verifiable outcome; and Bayesian Truth Serum and prediction markets are the most complex, requiring subjects to coordinate on the Bayesian Nash equilibrium (and assumptions such as common prior) while not requiring verifiable outcome. Based on our data and results, we do not claim that beliefs elicited using the proposed method are more accurate than those obtained via surveys or by methods that rely on a Bayesian Nash equilibrium and operate without verifiable outcomes. The purpose of the present study is to propose a workaround that makes moderately complex, incentive‑compatible methods applicable to the elicitation of health beliefs. In doing so, we address a gap for researchers who wish to collect health beliefs without completely abandoning truth‑telling incentives or adopting maximal theoretical and practical complexity.

## Supporting information

S1 Appendix Software Interface. (DOCX)

S2 Appendix Estimation procedure to recover subjective beliefs. (DOCX)

S3 Appendix Pre-experiment survey questionnaire. (DOCX)

S4 Appendix Post-experiment Survey. (DOCX)
