# Supplementary material for: Eliciting health beliefs: Difficulties and solutions
Source: PLoS One. 2026 May 28;21(5):e0347922. doi: 10.1371/journal.pone.0347922 (PMC13218472; doi:10.1371/journal.pone.0347922)
Supplement: S1 Appendix — (DOCX) [file pone.0347922.s001.docx]

**Appendix A: Software Interface**


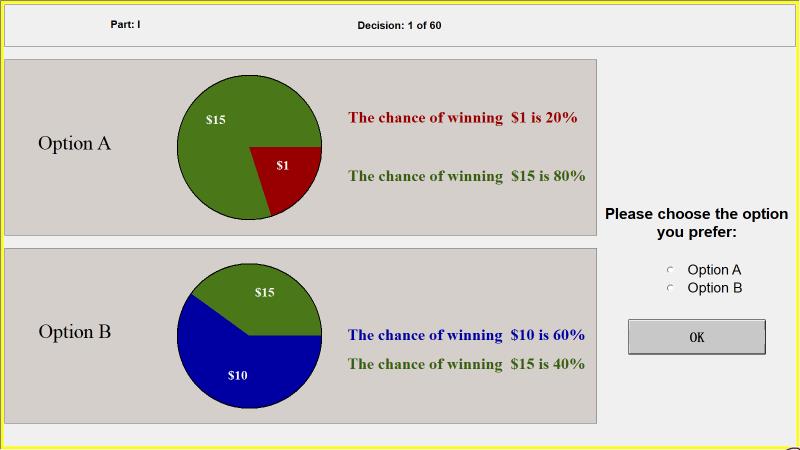


**Figure A1.** Example screenshot of the risk-preference task


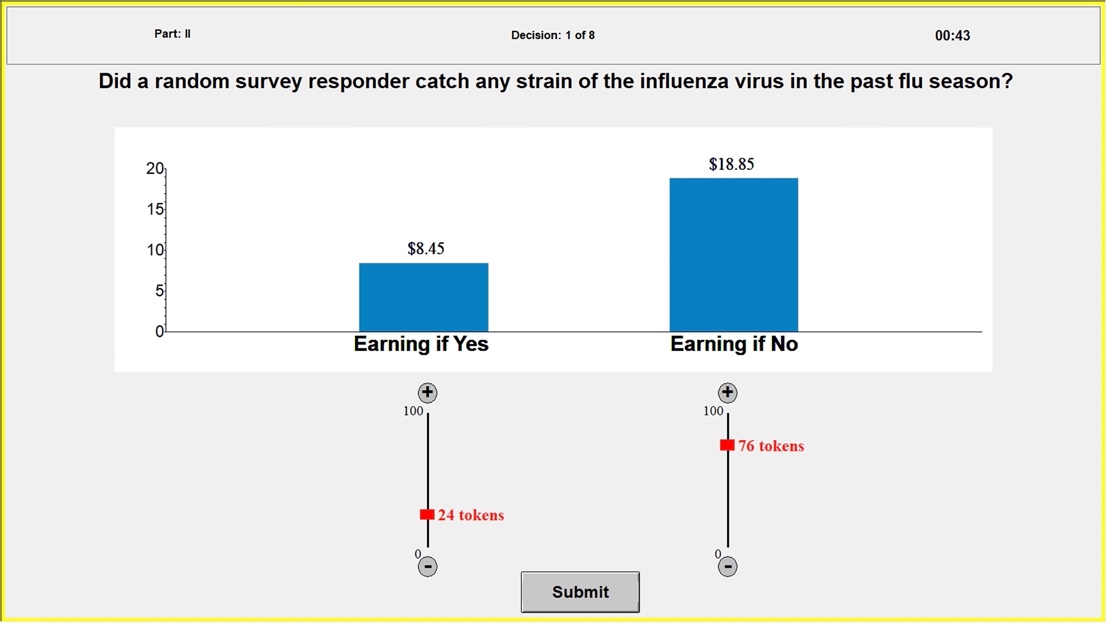
**Figure A2.** Interface to bet on whether a respondent contracted the flu


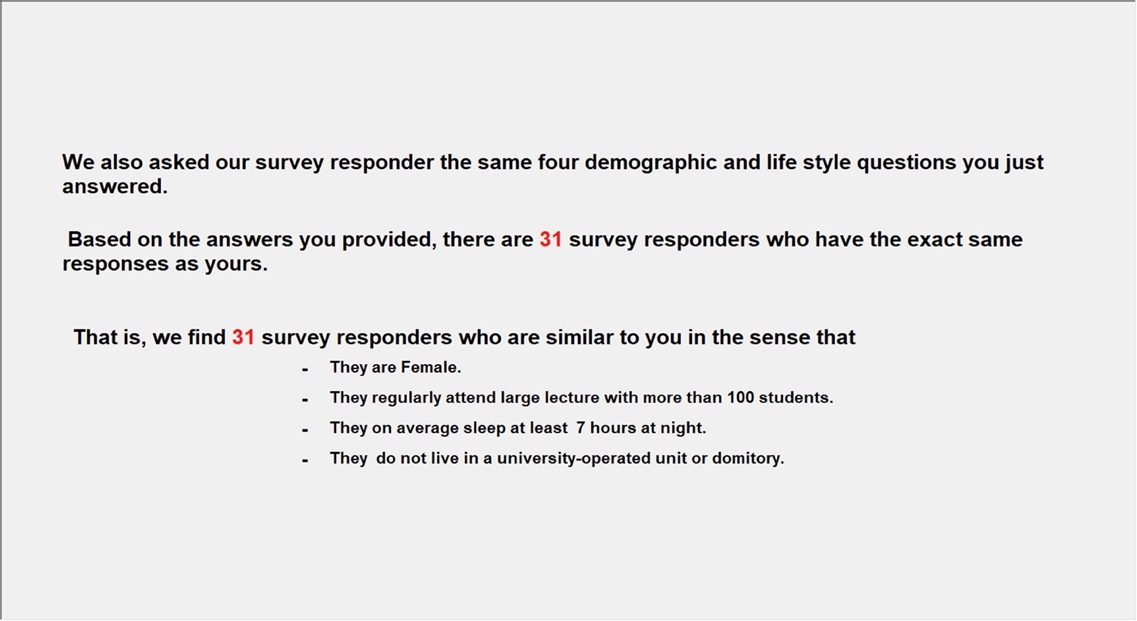


**Figure A3.** Information regarding the number of survey respondents in the matching subgroup


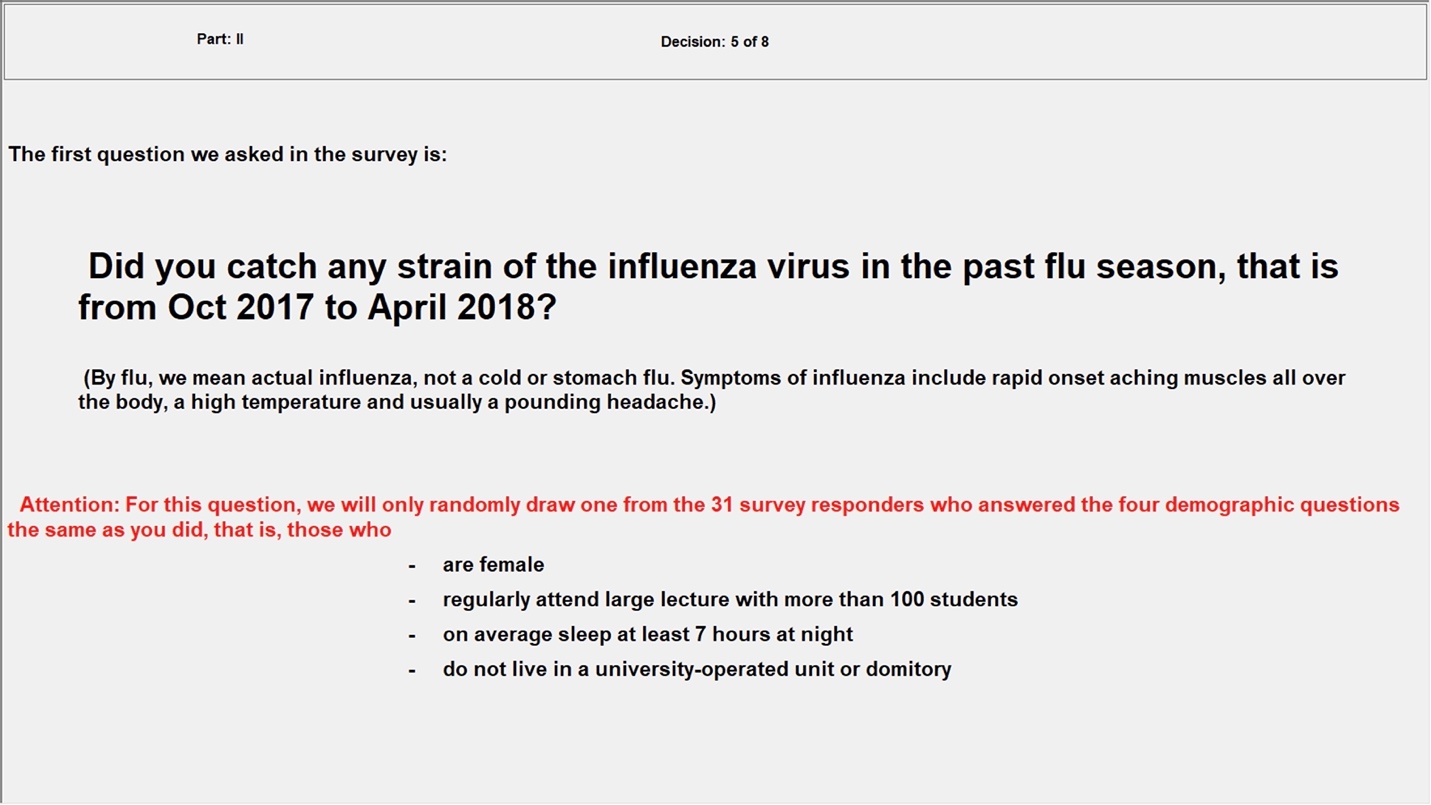


Figure A4. Leading page to the subgroup bet on the chance of flu


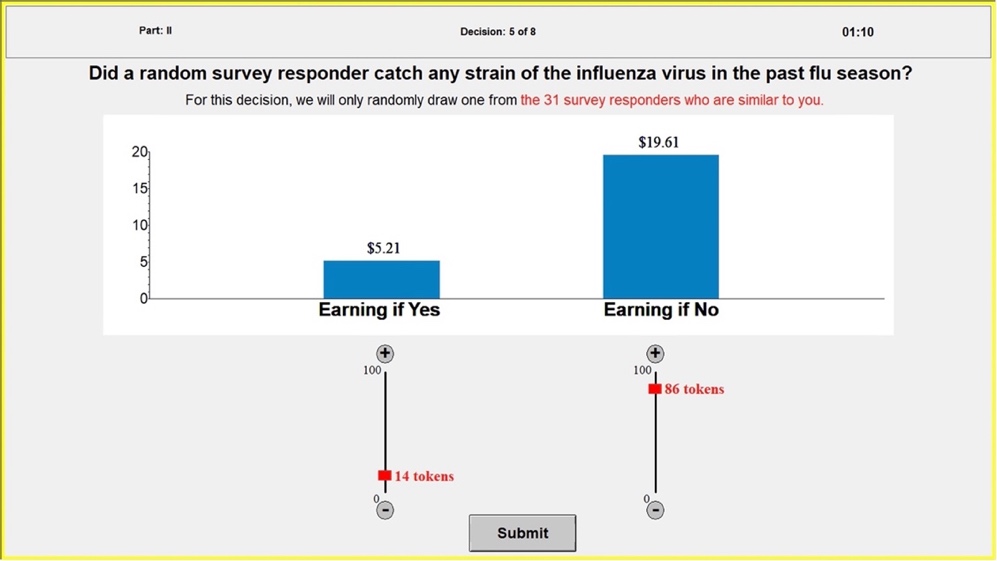


**Figure A5.** Interface to bet on whether a respondent with matching characteristics contracted the flu


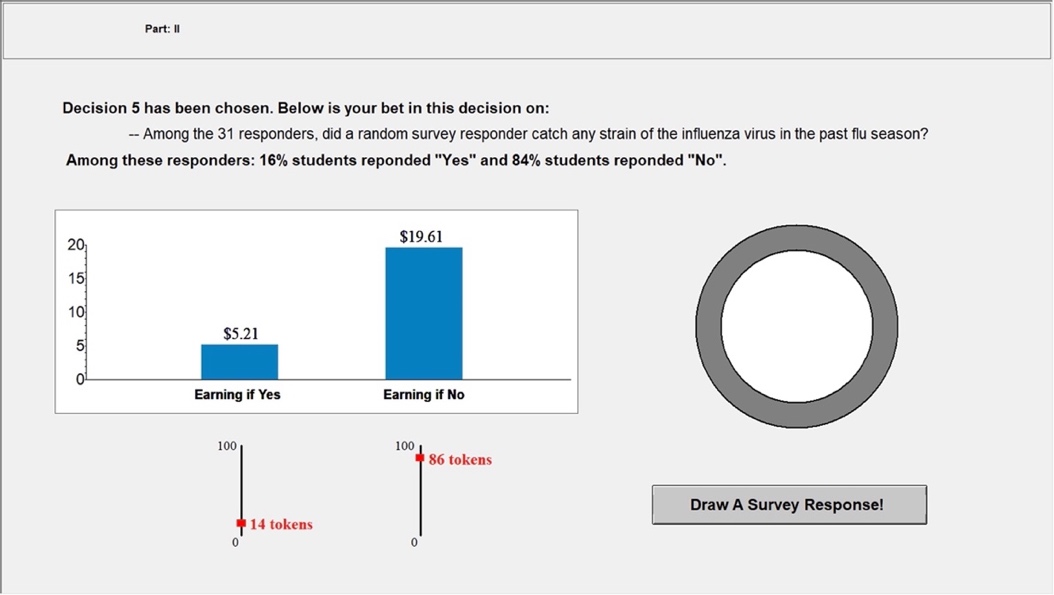


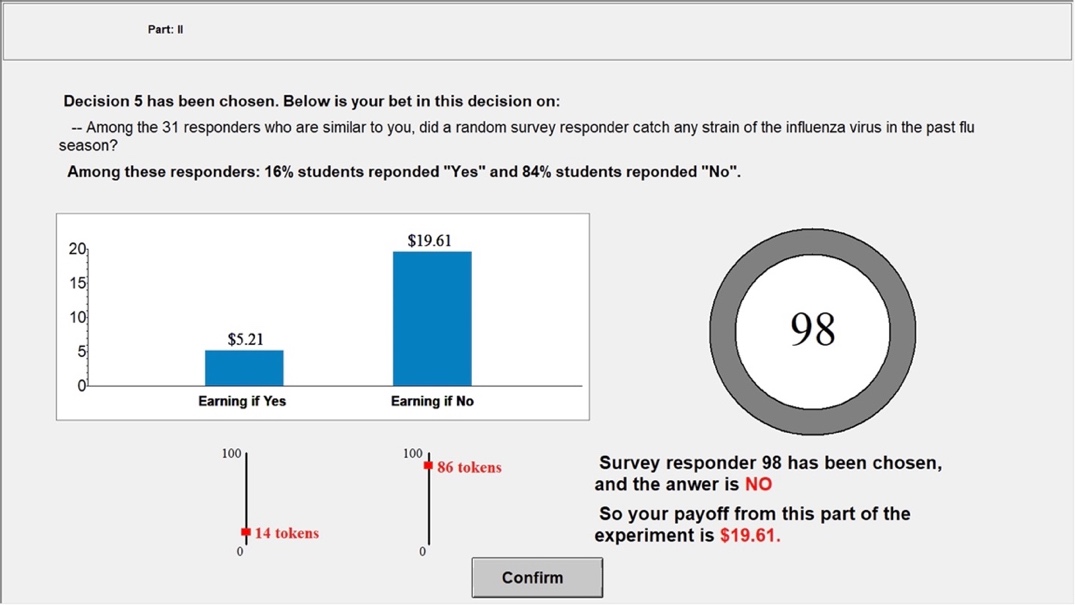


**Figure A6.** Determination of payoff in selected bet
