# Supplementary material for: Eliciting health beliefs: Difficulties and solutions
Source: PLoS One. 2026 May 28;21(5):e0347922. doi: 10.1371/journal.pone.0347922 (PMC13218472; doi:10.1371/journal.pone.0347922)
Supplement: S2 Appendix — (DOCX) [file pone.0347922.s002.docx]

**Appendix B: Estimation procedure to recover subjective beliefs**

Our target is to recover eight health beliefs for each subject: the probability of contracting flu for a general undergraduate student ($P_{1}$), the probability of taking a flu vaccine for a general undergraduate student ($P_{2}$), the probability of experiencing side effects after receiving a flu vaccine for a general undergraduate student ($P_{3}$), the probability of contracting flu after receiving a flu vaccine for a general undergraduate student ($P_{4}$), the probability of contracting flu of a student with matching characteristics ($P_{5}$), the probability of taking a flu vaccine of a student with matching characteristics ($P_{6}$), the probability of experiencing side effects after receiving a flu vaccine of a student with matching characteristics ($P_{7}$), and the probability of contracting flu after receiving a flu vaccine of a student with matching characteristics ($P_{8}$). Recall our experiment elicits each belief by asking a subject to bet on whether a random response drawn from the appropriate pre-experiment survey is "Yes" or "No" for the corresponding question. A subject splits 100 tokens between "Yes" and "No," and their monetary payoff when the random response is "Yes" or "No" is calculated according to the QSR formula. We refer to the percentage of tokens a subject allocates to "Yes" as their "report," following the tradition in this literature. Hence, we denote the eight reports as, $r_{1}, \ldots{, r}_{8}$, respectively.

The payoff each subject receives is:

$$\mathrm{Payoff}=\left\{ \begin{aligned} \$20-\$20\times\left( 1-r_{i} \right)^{2} , if the randomly selected response is "Yes" \\ \$20-\$20\times\left( 0-r_{i} \right)^{2}, if the randomly selected response is "No" \end{aligned} \right.$$

If subjects are risk-neutral and maximize their expected payoffs given their beliefs, the payoff structure ensures $r_{i}$ is equal to the underlying subjective probability $p_{i}$ for $i=1, . . . , 8$.^[[1]](#footnote-1)^ However, when a subject is not risk-neutral, $r_{t}$is a biased estimate of $p_{t}$. To mitigate this bias, we use a subject's risk preference to infer the underlying probabilities from their reports, as explained below.

We assume participants are characterized by the RDU model when evaluating uncertain prospects. A subject who reports $r_{i}$ for the $i^{th}$ bet, $i=1,\ldots, 8,$ receives the following RDU:

$$RDU_{i}(r_{i})=\pi_{YES}u\left( payoff if Yes \right)+\pi_{NO}u\left( payoff if No \right)=\pi_{YES} u\left( \$20-\$20\times\left( 1-r_{i} \right)^{2} \right)+\pi_{NO}u\left( \$20-\$20\times\left( 0-r_{i} \right)^{2} \right),$$

$\mathrm{where}\pi_{YES}$ and $\pi_{NO}$ are the decision weights associated with the two outcomes that add up to 1. Using the flexible Probability Weighting Function (PWF) proposed by[88]:

$$\omega\left( p \right)=\exp\left( -\eta\left( -\ln p \right)^{\phi} \right).$$

The decision weights are then calculated as follows:

- If $r_{i}\geq0.5$, then subjects receive a higher payoff when the outcome is Yes, applying flexible PWF suggested by [88]to the probability of Yes:

$$\pi_{YES}=\exp\left( -\eta\left( -\ln p_{i} \right)^{\phi} \right),$$

$$\pi_{NO}=1-exp \left( -\eta\left( -\ln p_{i} \right)^{\phi} \right),$$

- If $r_{i}<0.5$, then subjects receive a higher payoff when the outcome is No, applying PWF to the probability of No:

$$\pi_{NO}=\exp\left( -\eta\left( -\ln(1-p_{i} \right)^{\phi} \right),$$

$$\pi_{YES}=1-exp \left( -\eta\left( -\ln(1-p_{i} \right)^{\phi} \right)$$

$\mathrm{where} \eta>0$ and $\phi>0$ are the parameters that characterize a subject's probability weighting behavior. In addition, assume a subject evaluates the utility of a prize using a Constant Relative Risk Aversion (CRRA) coefficient utility function:

$$u\left( x \right)=\frac{\left( x \right)^{1-\alpha}}{1-\alpha}$$

where $\alpha$ is their Constant Relative Risk Aversion (CRRA) coefficient.

We allow subjects to choose reports in increments of 0.01$.$ The probability of observing $r_{i}$ is then:

$$\mathrm{Prob}\left( r_{i} \right)=\frac{exp[((RDU_{i} (r_{i} ))/v)/\mu_{r}]}{\sum_{R=0}^{1} exp[((RDU_{i} (R ))/v)/\mu_{r}]}$$

where R increases from 0 to 1 in increments of 0.01, ν is a normalizing term introduced in Wilcox (2011) and defined as the maximum utility over all possible reports minus the minimum utility over all possible reports, and $\mu_{r}>0$ is a Fechner noise parameter.

The likelihood of observing all eight reports of all subjects is then:

$$L^{\mathrm{Report}}\left( \alpha, \eta,\phi,P_{1},\ldots,P_{8},\mu_{r} \right)=\prod_{s=1}^{254} \prod_{i=1}^{8} \frac{exp[((RDU_{i} (r_{i} ))/v)/\mu_{r}]}{\sum_{R=0}^{1} exp[((RDU_{i} (R ))/v)/\mu_{r}]}.$$

Here $s=1,\ldots, 254$represent each subject and $r_{is}$ represent $i^{th}$ report of subject $s$. To recover $P_{i}$ from $r_{i}$, we use their binary lottery choices (BLR) to help us infer the RDU parameters $\alpha, \eta, \mathrm{and}\phi$. In the binary lottery tasks, subjects select a preferred lottery from a pair of lotteries. They do so for 40 pairs of lotteries. Each lottery is a probability distribution over prizes, where $p_{k}$ represents the probability of winning $\$x_{k}$, $k=1,2,3.$ The prizes are ranked from the highest to the lowest, such that $x_{1}\geq x_{2}\geq x_{3}$. A subject evaluates a lottery as:

$$RDU=\sum_{k=1,2,3} \pi_{k}u(x_{k})=\sum_{k=1,2,3} \pi_{k}\frac{\left( e+x_{k} \right)^{1-\alpha}}{1-\alpha}$$

where $e$is the endowment and $\pi_{k}$ is the decision weight associated with $\$x_{k}$, calculated as:

$$\pi\left( x_{1} \right)=\omega\left( p_{1} \right)$$

$$\pi\left( x_{2} \right)=\omega\left( p_{1}+p_{2} \right)-\omega\left( p_{1} \right)$$

$$\pi\left( x_{x} \right)=1-\omega\left( p_{1}+p_{2} \right)$$

Again, $\omega(.)$ takes the flexible PWF proposed by [88]:

$$\omega\left( p \right)=\exp\left( -\eta\left( -\ln p \right)^{\phi} \right).$$

where $t$ represents the $t^{th}$ lottery pair and denotes the two lotteries as left and right. We use $y_{t}=1$to represent a subject selecting the left lottery in pair $t$, $RDU^{L}$ to represent the utility of the left lottery, and $RDU^{R}$ to represent the utility of the right lottery.

The probability of observing $y_{t}=1$ is then:

$$\mathrm{Prob}\left( y_{t}=1 \right)=\frac{exp[\left( RDU^{L}/v \right){/\mu}_{b}]}{exp[\left( RDU^{L}/v \right){/\mu}_{b}]+exp[\left( RDU^{R}/v \right){/\mu}_{b}]}$$

where ν is a normalizing term for each lottery pair L and R, defined as the maximum utility over all prizes in this lottery pair minus the minimum utility over all prizes in this lottery pair, and $\mu_{b}>0$ is a Fechner noise parameter.

The likelihood of observing all 40 lottery choices of all subjects is then:

$$L^{\mathrm{Lottery}}\left( \alpha, \eta,\phi,\mu_{b} \right)=\prod_{s=1}^{254} \prod_{t=1}^{40} \left( \mathrm{Prob}\left( y_{t}=1 \right) \right)^{y_{ts}}\cdot\left( 1-\mathrm{Prob}\left( y_{t}=1 \right) \right)^{{1-y}_{ts}}$$

We can jointly maximize the two likelihoods below and estimate $\alpha, \eta,\phi,p_{1},\ldots,$ $p_{8}$ from their lottery choices and reports in the beliefs estimation tasks:

$$L^{\mathrm{Lottery}}\left( \alpha, \eta,\phi\right){\cdot L}^{\mathrm{Report}}\left( \alpha, \eta,\phi,P_{1},\ldots,P_{8} \right)$$

Note that the above specification ignores individual heterogeneity and assumes all subjects have the same parameters. We use *Stata ml* to estimate the parameters, and use the following logistic transformation in the likelihood functions to constraint $P_{i}$ to be between 0 and 1:

$$P_{i}=1-1\boldsymbol{/(}1\boldsymbol{+}\exp\left( p_{i} \right)\boldsymbol{),}$$

where $p_{i}\to-\infty, P_{i}\to0$ and $p_{i}\to+\infty, P_{i}\to1$. This is equivalent to

$$p_{i}=\ln\left( \frac{P_{i}}{1-P_{i}} \right).$$

For the three models reported in the paper, we introduce three sets of independent variables and model $p_{i}$ as a linear function of the independent variables:

$$p_{is}=\beta_{i0}+\boldsymbol{\beta}_{\boldsymbol{i}\boldsymbol{1}}\cdot\boldsymbol{X}_{\boldsymbol{is}}\boldsymbol{,}$$

where $s$ represents a subject and $\boldsymbol{X}_{\boldsymbol{is}}$ represents their characteristics. We then estimate $\beta_{i0}$ and $\boldsymbol{\beta}_{\boldsymbol{i}\boldsymbol{1}}$to see how $P_{i}$correlates with the vector of independent variables $X_{is}$. For Model 1 of Table 2, $\boldsymbol{X}_{\boldsymbol{is}}$ includes only the past relevant health outcomes of each subject. For Model 2 of Table 3, $\boldsymbol{X}_{\boldsymbol{is}}$consists of both relevant and irrelevant health outcomes of each subject in the past flu season.

While the above mentioned maximum likelihood estimation method allows us to correct for risk attitudes in subjects’ reports, it doesn’t lend itself for the use of fractional response regressions. As robustness checks, we run fractional response regression each report, without correcting for risk attitudes, as a function of $\boldsymbol{X}_{\boldsymbol{is}}$**.** We report the results in Tables B.3 and B.4. We also include the models in Tables 2 and 3 in these two tables as comparison. The results based on maximum likelihood method with corrections for risk attitudes and the results based on fractional regressions without corrections are qualitatively the same in terms of statistical significances. There are some expected quantitative differences due to the differences between the reports and estimated beliefs.

Table B.1. Probabilities and prizes in the 40 lottery pairs

* p<.05 ** p<.01 *** p<.001

Table B.2 Nuisance parameters for Table 2 and Table 3

| Elicited Belief | Own Health Outcome / Characteristics | Population | | Subgroup | |
| --- | --- | --- | --- | --- | --- |
|  |  | Model 1 | Model 1 Fractional | Model 1 | Model 1 Fractional |
| P Flu | Flu2017 | 1.025*** | 0.567*** | 1.147*** | 0.623*** |
|  |  | (0.207) | (0.115) | (0.248) | (0.134) |
|  | Constant | -0.820*** | -0.450*** | -0.907*** | -0.492*** |
|  |  | (0.0891) | (0.0456) | (0.0983) | (0.0489) |
| P Flu Shot | Flushot2017 | 0.643*** | 0.363*** | 0.786*** | 0.432*** |
|  |  | (0.152) | (0.0828) | (0.166) | (0.0865) |
|  | Constant | 0.264** | 0.144** | 0.167 | 0.0896 |
|  |  | (0.0997) | (0.0539) | (0.100) | (0.0534) |
| P Side Effect | No side effect after flu shot | -0.927*** | -0.507*** | -0.941*** | -0.497*** |
|  |  | (0.189) | (0.0983) | (0.232) | (0.115) |
|  | Side effect after flu shot | 1.390*** | 0.757*** | 0.755*** | 0.424*** |
|  |  | (0.267) | (0.127) | (0.228) | (0.126) |
|  | Constant | 0.183 | 0.0918 | 0.0298 | 0.0136 |
|  |  | (0.122) | (0.0637) | (0.135) | (0.0683) |
| P Flu After Shot | No flu after flu shot | -0.898*** | -0.360*** | -0.656* | -0.276** |
|  |  | (0.309) | (0.102) | (0.263) | (0.106) |
|  | Flu after flu shot | 0.308 | 0.185 | 0.364 | 0.220 |
|  |  | (0.660) | (0.309) | (0.634) | (0.301) |
|  | Constant | -1.352*** | -0.723*** | -1.264*** | -0.680*** |
|  |  | (0.134) | (0.0609) | (0.132) | (0.0615) |
| Observations | | 12,192 |  | 12,192 |  |

* p<.05 ** p<.01 *** p<.001

Table B.3 Comparison with Fractional Regression for Table 2

* p<.05 ** p<.01 *** p<.001

Table B.4 Comparison with Fractional Regression for Table 3

1. To be precise, risk-neutral subjects select their report, $r_{t}$, to solve the following problem:

$$\max_{r_{t}} p_{t}\cdot\left( \$20-\$20\times\left( 1-r_{t} \right)^{2} \right)+\left( 1-p_{t} \right)\cdot\left( \$20-\$20\times\left( 0-r_{t} \right)^{2} \right)$$

   where $p_{t}$ is their belief in the percentage of “Yes” responses to the corresponding question in the pre-experiment survey. The optimal solution is $r_{t}=p_{t}$. [↑](#footnote-ref-1)
