# Supplementary material for: Eliciting health beliefs: Difficulties and solutions
Source: PLoS One. 2026 May 28;21(5):e0347922. doi: 10.1371/journal.pone.0347922 (PMC13218472; doi:10.1371/journal.pone.0347922)
Supplement: S3 Appendix — (DOCX) [file pone.0347922.s003.docx]

**Appendix C: Pre-experiment survey questionnaire**

Q1: Are you a UMass student?

Yes or No

[If No, the entry is disqualified.]

[If Yes, continue]

Q2: Are you 18 years old and above?

Yes or No

[If No, the entry is disqualified.]

[If Yes, continue]

Q3: I would like to ask you to participate in a short survey. It includes 7-9 questions related to flu-shot decisions. Here is an information sheet that explains the purpose of the study and your rights as a participant. You can take this sheet with you once you have completed the survey. Are you willing to help me fill out the short survey?

[If No, move on to the next potential participant.]

[If Yes, continue]

Q4: What is the gender you identify with?

Q5. During this semester, on average, how many days per week do you do moderate-to-intense cardio or aerobic exercise for at least 30 minutes?

Q6. During this semester, do you regularly attend any large lectures with more than 100 students enrolled in the classroom? Yes or No

Q7. During this semester, on average, how many hours a night do you sleep?

Q8. Do you live in a University-operated housing unit or dormitory?

Q9: Did you catch any strain of the influenza virus in the past flu season, that is from Oct 2017 to April 2018? (By flu, we mean actual influenza, not a cold or stomach flu. Symptoms of influenza include rapid onset, aching muscles all over the body, high body temperature, and usually a pounding headache.)

Q10: Did you take a flu shot in the past flu season?

If Q10 is Yes,

Q11: Did you experience any side effects immediately after taking the flu shot? (The side effects may include any of the following: soreness, redness, or swelling where the shot was given • hoarseness • sore, red, or itchy eyes • cough • fever • aches • headache • itching • fatigue • seizure)

Q12: Did you catch any strain of the influenza virus after taking the flu shot? (By flu, we mean actual influenza, not a cold or stomach flu. Symptoms of influenza include rapid onset, aching muscles all over the body, high body temperature, and usually a pounding headache.)
