# Supplementary material for: Eliciting health beliefs: Difficulties and solutions
Source: PLoS One. 2026 May 28;21(5):e0347922. doi: 10.1371/journal.pone.0347922 (PMC13218472; doi:10.1371/journal.pone.0347922)
Supplement: S4 Appendix — (DOCX) [file pone.0347922.s004.docx]

**Appendix D: Post-experiment Survey**

Part I. Information on flu shot decisions

1. Did you catch any strain of the influenza virus in the past flu season, that is from Oct 2017 to April 2018?
2. Did you take a flu shot in the past flu season from Oct 2017 to April 2018?
3. If your answer to the previous question is Yes, please check the service provider for your flu shot in the past flu season from Oct 2017 to April 2018 below

- I did not take a flu shot
- University Health Services at UMass
- Commercial pharmacy or Urgent Care Clinic
- My primary care provider and other

1. Did you experience any side effects immediately after the flu shot in the past flu season from Oct 2017 to April 2018?
2. Did you catch any strain of the influenza virus after the flu shot in the past flu season from Oct 2017 to April 2018?
3. Did you catch any strain of the influenza virus in the past flu season, that is from Oct 2016 to April 2017?
4. Did you take a flu shot in the past flu season from Oct 2016 to April 2017?
5. If your answer to the previous question is Yes, please check the service provider for your flu shot in the past flu season from Oct 2016 to April 2017 below

- I did not take a flu shot
- University Health Services at UMass
- Commercial pharmacy or Urgent Care Clinic
- My primary care provider and other

1. Did you experience any side effects immediately after the flu shot in the past flu season from Oct 2016 to April 2017?
2. Did you catch any strain of the influenza virus after the flu shot in the past flu season from Oct 2016 to April 2017?
3. Do you plan to take a flu shot in this flu season, that is from Oct 2018 to April 2019?
4. Please check the service provider you plan to get the flu shot with

- I will not take a flu shot
- University Health Services at UMass
- Commercial pharmacy or Urgent Care Clinic
- My primary care provider and other

Part II. General Information

1. What year were you born? ___________
2. What is your biological gender at birth?

- Male
- Female
- Other ____________________

1. What gender are you identifying with?

- Male
- Female
- Other ____________________

1. Please specify your ethnicity origin (or Race):

- White
- African American
- Hispanic or Latino
- Native American or American Indian
- Asian/Pacific Islander
- Mixed Origins/Races
- Other: ____

1. Which academic cohort do you belong to as of this Fall semester?

- Freshman
- Junior
- Sophomore
- Senior
- Graduate student

1. How many classes are you enrolled in during this Fall semester? __________
2. Which school are you majored in?

- College of Education
- College of Engineering
- College of Humanities and Fine Arts
- College of Information and Computer Sciences
- College of Natural Sciences
- College of Nursing
- College of Social and Behavioral Sciences
- Commonwealth Honors College
- Isenberg School of Management
- Undetermined

1. What is your current GPA? ______________
2. On average, how many hours do you work every week (including both paid or volunteer work)? __________
3. Are you an athlete?
4. If your answer to 11 is yes, which type of sport do you play? __________
5. Typically, how often do you exercise?

0-1 times a week

2-3 times a week

4-5 times a week

more than 6 times a week

1. Do you smoke?
2. Have you ever smoked in the past?
3. What is your height (enter in feet and inches)
4. What is your weight (enter in pounds, lbs.)
5. In general, would you say that your behavior and the decisions you make often involve high risk? (7--strongly agree; 6--Agree; 5--somewhat agree, 4--indifferent, 3--somewhat disagree, 2--disagree, 1--strongly disagree).
6. In general, would you say that your behavior and the decisions you make show that you are a patient person? (7--strongly agree; 6--Agree; 5--somewhat agree, 4--indifferent, 3--somewhat disagree, 2--disagree, 1--strongly disagree).

(Please indicate your level of trust below on a scale of 0 to 10. A higher number indicates greater trust.)

1. Recall in Part I of the study, you were asked to choose between two pie graphs representing chances of winning different prizes. When you were making these decisions, how much do you trust that the rotating pie stops at a truly random location?
2. Recall in Part II of the study, you were asked to bet on how a group of UMass students have answered in a flu-fact survey. When you were making these bets, how much do you trust that the response you drew to determine your payoff was truly random from the relevant survey pool?
3. In Part II, when you were making these bets, how much do you trust that the researchers actually conducted the flu-fact surveys in Spring 2018 with UMass students and are using the results to determine your payoff?
4. In Part II, how much do you trust that the survey participants interviewed in Spring 2018 answered the flu-fact questions truthfully?
5. Recall in Part III of the study, you were asked to choose between payment options with different amounts of time delay. When you were making these decisions, how much do you trust that the research team will actually deliver the future payments to those who are entitled to it?
6. What is the best email address that we can reach you in the future?
7. Have you ever participated in any economic experiments before?
8. Have you ever participated in any experiment where your earning is determined by drawing balls from a box/bingo cage, or rolling dice?
